# MULTILEVEL FACTORS ASSOCIATED WITH RACIAL AND ETHNIC DISPARITIES IN TIMELY DIAGNOSIS OF DEMENTIA IN US OLDER ADULTS

**DOI:** 10.1093/geroni/igae098.1496

**Published:** 2024-12-31

**Authors:** Yuting Qian, Fan Li, Xi Chen

**Affiliations:** Yale University, New Haven, Connecticut, United States; Yale University, New Haven, Connecticut, United States; Yale University, New Haven, Connecticut, United States

## Abstract

In the U.S., non-Hispanic Black and Hispanic older adults are disproportionately more likely to develop dementia but less likely to receive a timely diagnosis than White older adults. Quality care for people with dementia starts with an early diagnosis, a key goal of the national plan to address dementia. This study examines individual-level and neighborhood-level factors associated with racial and ethnic differences in timely dementia diagnosis. We used 1998-2020 Health and Retirement Study (HRS) data, which contains detailed information on cognitive assessments and provider diagnoses for a representative sample of U.S. older adults. This was then linked to the National Neighborhood Data Archive (NaNDA) for neighborhood contexts. We find that racial and ethnic disparities in timely dementia diagnosis were substantially attenuated after adjusting for individual-level and neighborhood-level factors. The adjusted odds ratio of having a timely dementia diagnosis increased from 0.60 (95% CI: 0.48-0.76) to 0.76 (95% CI: 0.58-1.00) for non-Hispanic Black older adults compared to their White counterparts. A mediation analysis revealed that household income and education explained the largest proportion of the disparity between non-Hispanic Black and White older adults (17.9% and 12.7%, respectively), followed by neighborhood affluence (10.9%), neighborhood disadvantage (9.9%), and health care access (measured by physician density and other health practitioner density). These patterns were similar for Hispanic compared to White older adults. Our findings may inform targeted interventions and policies that emphasize socioeconomic risk factors and health service availability to reduce racial and ethnic disparities in timely dementia diagnosis and promote health equity.

